# Medroxyprogesterone acetate, unlike norethisterone, increases HIV-1 replication in human peripheral blood mononuclear cells and an indicator cell line, via mechanisms involving the glucocorticoid receptor, increased CD4/CD8 ratios and CCR5 levels

**DOI:** 10.1371/journal.pone.0196043

**Published:** 2018-04-26

**Authors:** Michelle F. Maritz, Roslyn M. Ray, Alexis J. Bick, Michele Tomasicchio, John G. Woodland, Yashini Govender, Chanel Avenant, Janet P. Hapgood

**Affiliations:** 1 Department of Molecular and Cell Biology, University of Cape Town, Cape Town, South Africa; 2 Centre for Lung Infection and Immunity, Division of Pulmonology and UCT Lung Institute, Department of Medicine, University of Cape Town, Cape Town, South Africa; 3 Institute of Infectious Diseases and Molecular Medicine, University of Cape Town, Cape Town, South Africa; University of Texas Rio Grande Valley, UNITED STATES

## Abstract

High usage of progestin-only injectable contraceptives, which include the intramuscular injectables depo-medroxyprogesterone acetate (DMPA-IM, Depo-Provera) and norethisterone (NET) enanthate (NET-EN or Nur-Isterate), correlates worldwide with areas of high HIV-1 prevalence. Epidemiological data show a significant association between usage of DMPA-IM and increased HIV-1 acquisition but no such association from limited data for NET-EN. Whether MPA and NET have similar effects on HIV-1 acquisition and pathogenesis, and the relationship between these effects and the dose of MPA, are critical issues for women’s health and access to suitable and safe contraceptives. We show for the first time that MPA, unlike NET, significantly increases HIV-1 replication in peripheral blood mononuclear cells (PBMCs) and a cervical cell line model. The results provide novel evidence for a biological mechanism whereby MPA, acting via the glucocorticoid receptor (GR), increases HIV-1 replication by at least in part increasing expression of the CCR5 HIV-1 coreceptor on target T-lymphocytes. MPA, unlike NET, also increases activation of T-cells and increases the CD4/CD8 ratio, suggesting that multiple mechanisms are involved in the MPA response. Our data offer strong support for different biological mechanisms for MPA versus NET, due to their differential GR activity. The dose-dependence of the MPA response suggests that significant effects are observed within the range of peak serum levels of progestins in DMPA-IM but not NET-EN users. Dose-response results further suggest that effects of contraceptives containing MPA on HIV-1 acquisition and disease progression may be critically dependent on dose, time after injection and intrinsic factors that affect serum concentrations in women.

## Introduction

Understanding the differential mechanisms of action and dose-dependent effects of the progestins medroxyprogesterone acetate (MPA) and norethisterone (NET) and effects on HIV-1 pathogenesis are crucial to women’s health. The most common form of contraception in developing countries is the three-monthly intramuscular injection of 150 mg of MPA (Depo-Provera or DMPA-IM), while NET enanthate (Nur-Isterate or NET-EN), a two-monthly injection of 200 mg of NET-EN, is less widely used in developing countries. A three-monthly subcutaneous formulation of DMPA (DMPA-SC marketed as Sayana^®^ Press), with a 30% lower dose (104 mg), is currently being introduced worldwide. Epidemiological data suggest a significant 1.4-fold increased risk of HIV-1 acquisition for DMPA-IM users compared to no hormonal contraception, although the data may be confounded by behavioural factors [1–3], while no such association is shown for limited data on NET-EN, and no information is available for DMPA-SC and HIV-1 acquisition risk [1]. Determination of the absolute and relative risk factors for HIV-1 acquisition and biological mechanisms for DMPA-IM, DMPA-SC and NET-EN is a critical issue for women’s health, especially in Sub-Saharan Africa [4–7].

Although the mechanisms whereby DMPA-IM may increase HIV-1 acquisition in the female genital tract are currently unclear, there is mounting evidence from clinical, animal and *in vitro* data to suggest multiple mechanisms [8, 9]. While the dose-dependence of these effects is unclear, recent data suggest that time after injection with DMPA-IM [9], corresponding to varying MPA serum concentrations, may be critical. There are no clinical or animal data on possible biological mechanims relevant to HIV-1 pathogenesis for DMPA-SC or NET-EN, while limited *ex vivo* data suggest that NET has no effect on immune function, unlike MPA [10–15]. Whether physiologically significant concentrations of MPA directly affect replication of infectious HIV-1 virus in target cells is unclear from the literature, while no information is available for NET [16, 17]. MPA may directly affect HIV-1 coreceptor expression levels in HIV-1 target cells, as is suggested from one report [16], while the effects of NET are unknown. Interestingly, progesterone did not increase CCR5 expression in non-activated PBMCs, but decreased IL2-induced CCR5 expression in activated PBMCs, which was accompanied by a slight resistence to HIV infection [18].

MPA, NET and progesterone differ in their glucocorticoid-like properties and are shown to exert very different biological responses via the glucocorticoid receptor (GR) [10–14, 19, 20]. Designed to act via the progesterone receptor (PR), progestins act to varying degrees via other members of the steroid receptor family of proteins [20–24]. These include the androgen, glucocorticoid, mineralocorticoid, and estrogen receptors (AR, GR, MR and ER, respectively). MPA is an outlier amongst this group of progestins, since it binds to the GR with a relatively high affinity and acts like a full to partial GR agonist, depending on cellular context, while NET exhibits almost no GR activity. Dose-response analysis defines the potency of a steroid response, or EC_50_, as the concentration required for half maximal activity [19, 25]. The EC_50_ or potency for MPA regulation of gene expression via the GR in *ex vivo* cell models varies in different T-cells and for different genes and occurs in the range of about 1–100 nM [10–13, 26–28], while NET has no GR activity [8, 10–13]. The MPA potencies *ex vivo* fall within the range of peak serum levels (C_max_) of MPA in DMPA-IM users [8, 19].

Given the clinical data suggesting that MPA but not NET increases HIV-1 acquisition, we sought to investigate the dose dependence and direct effects of both MPA and NET on R5 HIV-1 replication in PBMCs. Towards further understanding the biological mechanisms of these potential effects, we investigated the role of the GR and CCR5 in both PBMCs and an indicator cell line.

## Materials and methods

### Compounds, antibodies and plasmids

(11b,16a)-9-Fluoro-11,17,21-trihydroxy-16-methylpregna-1,4-diene-3,20-dione (dexamethasone; Dex, D4902), 6α-methyl-17α-hydroxy-progesterone acetate (medroxyprogesterone acetate; MPA, M1629), 4-pregnene-3,20-dione (progesterone; P4, P0130), 17α-ethynyl-19-nortestosterone (norethisterone; NET, N4128), and 11β-(4-dimethylamino)phenyl-17β-hydroxy-17-(1propynyl)estra-4,9-dien-3-one (Mifepristone; RU486, M8046) were purchased from Sigma-Aldrich, South Africa. Interleukin 2 (IL2) and maraviroc (MVC) were obtained through the AIDS Research and Reference Reagent Program, Division of AIDS, NIAID, NIH. 3-(4,5-Dimethylthiazol-2-yl)-2,5-diphenyltetrazolium bromide (MTT, M5655) was purchased from Sigma-Aldrich, South Africa. Antibodies to glucocorticoid receptor (GR) (H-300, sc-8992), androgen receptor (AR) (441, sc-7305), mineralocorticoid receptor (MR) (H-300, sc-11412, as well as C-19, sc-6861), estrogen receptor alpha (ERα) (MC-20, sc-542) and GAPDH (0411; sc-47724) were obtained from Santa Cruz Biotechnology, USA. Antibodies to the progesterone receptor (PR) (NCL-LPGR-312) were purchased from Leica Biosystems (Novocastra, United Kingdom). Secondary antibodies for primary detection were purchased from Santa Cruz Biotechnology, USA, and include anti-mouse (sc-2005) and anti-rabbit (sc-2313). Anti-CD3 fluorescein isothiocyanate (FITC), (300440), anti-CD4 phycoerythrin-Dazzle 594 (PE-Dazzle 594) (357412), anti-CD8 PE/Cy5 (300910), anti-CD25 PE (356104), anti-CD69 PE/Cy7 (310912), anti-CCR5 allophycocyanin (APC) (359122) and ZOMBIE NIR (423113) were purchased from Biolegend (USA). An R5 infectious molecular clone that had a luciferase gene inserted adjacent to the *env* gene in the HIV-1 NL4-3 backbone known as NL—LucR.T2A—BaL.ecto, was a kind gift from by Dr. Christina Ochsenbauer [29], and known as HIV-1_BaL-Renilla_ in this study.

### Cell culture

Human embryonic kidney cells (HEK293T) were purchased from America Type Culture Collection (ATCC, USA). Human cervical TZM-bl cells were procured from the NIH AIDS Reagent Program, Division of AIDS, NIAID, NIH from Dr. John C. Kappes, Dr. Xiaoyun Wu and Tranzyme Inc. (ARP, NIH, USA). Cells were cultured in 75 cm^2^ flasks (Greiner Bio-one International, Austria) in Dulbecco’s modified Eagle’s medium [(DMEM) (Sigma-Aldrich, South Africa) supplemented with 1 mM sodium pyruvate (58636, Sigma-Aldrich, South Africa), 44 mM sodium bicarbonate (Sigma-Aldrich, South Africa), 10% (v/v) foetal bovine serum (Thermo Scientific, South Africa) 100 IU/mL penicillin and 100 mg/mL streptomycin (P4333, Sigma-Aldrich, South Africa); full DMEM]. All cells were maintained at 37°C in a water jacketed incubator (90% humidity and 5% CO_2_). Cells were passaged twice a week, with 0.25% (w/v) trypsin/0.1% (w/v) EDTA in PBS (Sigma-Aldrich, South Africa). Trypsinisation was terminated with neutralisation medium (full DMEM). All cells were routinely tested and found to be mycoplasma-free.

### Virus propagation

Initial viral stocks were prepared as previously described [30] with a few modifications. HEK293T cells were seeded at a density of 4 X 10^6^ cells in a 10 cm^2^ plate in full DMEM supplemented with 25 mM HEPES buffer (Lonza, Germany) at 37°C in a water-jacketed incubator (90% humidity and 5% CO_2_). The next day, media was replaced and cells were transfected with 12 μg HIV-1_BaL-Renilla_ or a control (DMEM) using X-tremeGENE 9 DNA transfection reagent (Roche Applied Science, South Africa) according to the manufacturer’s specifications. Cells were incubated for 48 hours at 37°C, the medium was passed through a 0.22 μM filter and charcoal-stripped (c-s) FCS (Thermo Scientific, USA) was added to a final concentration of 12.5%. The viral stocks were aliquoted and stored at -80°C until use. Virus titres were determined using the TZM-bl assay as previously described [29]. Cells were harvested 72 hours later with 120 μL Bright-Glo luciferase lysis buffer (Promega, USA). Fluorescence was determined on a luminometer (Modulus Microplate, Promega, USA), where relative light units (RLU) were measured for each well. The titre of the virus stock was determined using the Reed Muench method and expressed as log infectious units (IU)/mL [31].

### PBMC isolation and infection assay

Permission to perform these studies was granted by the Human Research Ethics Committee of the Faculty of Health Sciences of the University of Cape Town (approval number: HREC 210/2011). Buffy packs were obtained from anonymous healthy female donors who were negative for HIV-1, syphilis and hepatitis B and C from the Western Province Blood Transfusion Services, after written informed consent. PBMCs were isolated using Histopaque (H1077 Hybri-MaxTM; Sigma-Aldrich, South Africa) density centrifugation with Leucosep tubes (Greiner Bio-One, Germany) according to the manufacturer’s instructions. The isolated PBMCs were washed twice with PBS supplemented with 1% (v/v) c-s FCS (Thermo Scientific, South Africa). PBMCs were subsequently cultured in high glucose (4.5 g/mL) RPMI 1640 (Lonza, Switzerland) with 10% (v/v) c-s FCS (Thermo Scientific, South Africa), 2 mM L-glutamine (G7513, Sigma-Aldrich, South Africa), 100 IU/mL penicillin and 100 mg/mL streptomycin (Sigma-Aldrich, South Africa) and 30 U/mL IL2 at 37°C in a water-jacketed incubator (90% humidity and 5% CO_2_).

After stimulation with ligands, PBMCs were infected with 10 IU/mL HIV-1BaL-Renilla or with a mock infection control (RPMI only), in the presence of the hormones, for 2 hours at 37°C. PBMCs were washed 4 times with 1 X PBS supplemented with 1% cs-FCS. Thereafter, full RPMI with IL2, was added and PBMCs were incubated for a further 5 days at 37°C. PBMCs were harvested at day five post-infection for *Renilla* luciferase expression using *Renilla* luciferin (Promega, USA), according to manufacturer’s instructions. Luminescence was determined on a luminometer (Modulus Microplate, Promega, USA), where RLU were measured for each well. Viability was measured using the MTT assay and measured on a spectrophotometer (Thermo Scientific, USA) at 595 nm. PBMCs remained viable for the duration of the infection assay as determined by the MTT assay. Infection was calculated by dividing the RLU obtained for each sample by the average MTT absorbance value for that sample group. Thereafter, relative infection was calculated by setting vehicle control (EtOH) to 100% infection.

### TZM-bl infection assay

TZM-bls were seeded at a concentration of 5 X 10^4^ cells/mL in a 96-well flat bottomed culture plate in full DMEM. The following day the TZM-bl cells were either stimulated with hormone or maraviroc (MVC) for 24 hours in triplicate. Cells were then infected with 20 IU/mL HIV-1_BaL_Renilla_ and were harvested 48 hours later with Bright-Glo luciferase lysis buffer (Promega, USA). Luminescence was determined on a luminometer (Modulus Microplate, Promega, USA), where relative light units were measured for each well. Viability was measured using the MTT assay and measured on a spectrophotometer (Thermo Scientific, USA) at 595 nm. Luciferase readings were normalized to MTT values (RLU/MTT). Relative infection was calculated by setting the vehicle control (EtOH) to 100% relative infection.

### Knockdown of GR by siRNA

TZM-bl cells were seeded at 1 X 10^5^ cells/well in 12-well plates. After 24 hours, cells were transfected with 10 nM non-silencing control (NSC) or siRNA targeting the human GR (siGR, Qiagen, South Africa) for 48 hours. Cells were re-seeded at a concentration of 2 X 10^5^ cells/mL in a 12-well culture plate (for western blotting) or at a concentration of 2.5 X 10^4^ cells/well into 96-well plates in quadruplicate wells for another 24 hours, followed by stimulation for 24 hours with 100 nM MPA or vehicle (EtOH). Media was removed and replaced with phenol red-free DMEM containing 10 IU/mL HIV-1_BaL-Renilla_ (HIV-BaL) or equivalent volume of virus control. Western blot samples were washed once with PBS and lysed with 50 μl 2 X SDS sample buffer (5 X SDS sample buffer: 100 mM Tris-Cl pH 6.8, 5% (w/v) SDS, 20% (v/v) glycerol, 2% ß-mercaptoethanol and 0.1% (w/v) bromophenol-blue) and boiled for 10 minutes at 100°C. The infected cells were harvested 72 hours later for luciferase (infection) and for cell viability using the MTT assay, as described above.

### RNA isolation and real time quantitative PCR (qPCR)

TZM-bl cells were seeded at a concentration of 2 X 10^5^ cells/mL in a 12-well culture plate in full DMEM. The following day the TZM-bl cells were stimulated with hormone for 24 hours, harvested in 400 μl TriReagent^®^ (T9424, Sigma-Aldrich, South Africa) and processed for RNA according to the manufacturer’s instructions.

250 ng total RNA was reverse-transcribed using the Transcriptor First Strand Synthesis cDNA kit (Roche Applied Science, South Africa) according to the manufacturer’s instructions. cDNA samples were stored at -80°C until use in subsequent real time qPCR reactions. Real-time quantitative PCR (qPCR) was performed using the Bioline SensiMix^™^ SYBR^®^ no ROX kit (QT650-05, Bioline USA) on a RotorGene 3000 (Qiagen, Netherlands) real time qPCR machine, according to manufacturer’s instructions. The steroid receptor primers and profiles were previously established [14]. CCR5 and CXCR4 were amplified with the following primer pairs 5’ TGGACCAAGCTATGCAGGTG 3' and 5' CGTGTCACAAGCCCACAGAT 3' and CXCR4 5'GAAATGGGCTCAGGGGACTAT 3' and 5' TTCAGCCAACAGCTTCCTTGG 3' with a Ta of 55°C and 60°C respectively. CD4 primers and profile were as previously reported [32] while for GAPDH the primers were by Verhoog et al. (2011) [33]. Relative transcript levels were determined using the method as previously described [34], with the vehicle control set to 1.

### Flow cytometry

Flow cytometry was performed as described previously [14] with a few modifications. Two million PBMCs (at a concentration of 2 million/mL) in full RPMI were placed into 5 mL Becton Dickinson Falcon tubes (BD Scientific, South Africa). PBMCs were subsequently stimulated with ligands or vehicle for 24 hours or 7 days at 37°C in a water jacketed incubator. After treatment, PBMCs were stained with anti-CD3 FITC, anti-CD4 PE-DAZZLE 594, anti-CD8 PE/Cy5, anti-CD25 PE or anti-CD69 PE/Cy7 and anti-CCR5 APC antibodies and the viability dye, ZOMBIE NIR (Biolegend, USA) at room temperature for 15 minutes in the dark. After staining, PBMCs were washed with PBS and resuspended in 1 X Cell Fix solution (Becton-Dickinson, USA). Samples were acquired using a BLSRII Becton-Dickinson flow cytometer (Becton-Dickinson, USA) and analysed using Flow Jo software (version 10.1, Treestar, Inc, Ashland, Ore). Lymphocytes were gated according to their forward- and side-scatter profiles. Only the single cellular population was analysed. There was no difference in the viability of PBMCs between treatments, as assessed by ZOMBIE NIR or MTT and PBMCs remained >90% viable for the duration of the experiments. Dead cells were excluded from the scatter plots prior to analysis and negative gates were set using minus fluorescence one (MFO) controls. Results are represented as either frequency (as a percentage of total) or expression (median fluorescence intensity, MFI). Relative fold change in frequency or expression levels was calculated by setting vehicle control (EtOH) expression to 1.

### Western blotting

TZM-bl western blot samples were obtained as described above (Knockdown of GR by siRNA). For the steroid receptor positive controls, COS-1 cells were seeded into 12-well plates (Greiner bio-one, Cellstar) at a density of 25 X 10^4^ cells/well. The next day the cells were transfected with 1 μg/well of empty vector, GR, AR, or PR or empty vector and 2 μg/well of MR or ERα using FuGENE^™^ 6 (Roche Applied Science Diagnostics, South Africa) and incubated for another 24 hours, before being harvested as described above. Western blotting was performed as previously described [35].

### Statistical analysis

Results were analysed using GraphPad PRISM (version 6) software from GraphPad Software Inc (La Jolla California, USA). Data were tested for normality before parametric tests were performed using the D’Agostino-Pearson omnibus normality test for large data sets and the Kolmogorov-Smirnov test with Dallal-Wilkinson-Lillefor P value for small data sets (n < 6). For parametric data, where samples were treated with ligands at one time point, a one-way ANOVA, with either a Dunnett’s or Tukey’s multiple comparisons post-test, comparing each group to control or each other was performed. For comparison between two conditions, an unpaired two-tailed student’s t-test was performed. For dose-response curves, data were analysed with the maximal response set to 100% and a non-linear regression model was employed, plotting log agonist vs response, with the Hill slope set to 1. For data that were non-parametric, a Kruskal-Wallis ANOVA with Dunn’s multiple comparisons test was performed when comparing samples to each other. Additionally, when two groups were compared to each other, a non-parametric Mann-Whitney test (where the vehicle control was not normalized to 1) or Wilcoxon signed rank test (where the vehicle control was normalized to 1) was performed. Data were expressed as mean ± SEM on histograms or XY scatter charts, with n values given in each figure legend. Where statistical significance of difference was obtained relative to a single control statistical significance is denoted by *, **, ***, or **** to indicate p<0.05, p<0.01, p<0.001, or p<0.0001 respectively. Where statistical significance of difference was obtained between two values, this is indicated with lines between the two sample sets.

## Results

### MPA, unlike NET, increases HIV-1 replication in PBMCs at peak serum levels of DMPA users, via the GR

**Fig 1:**
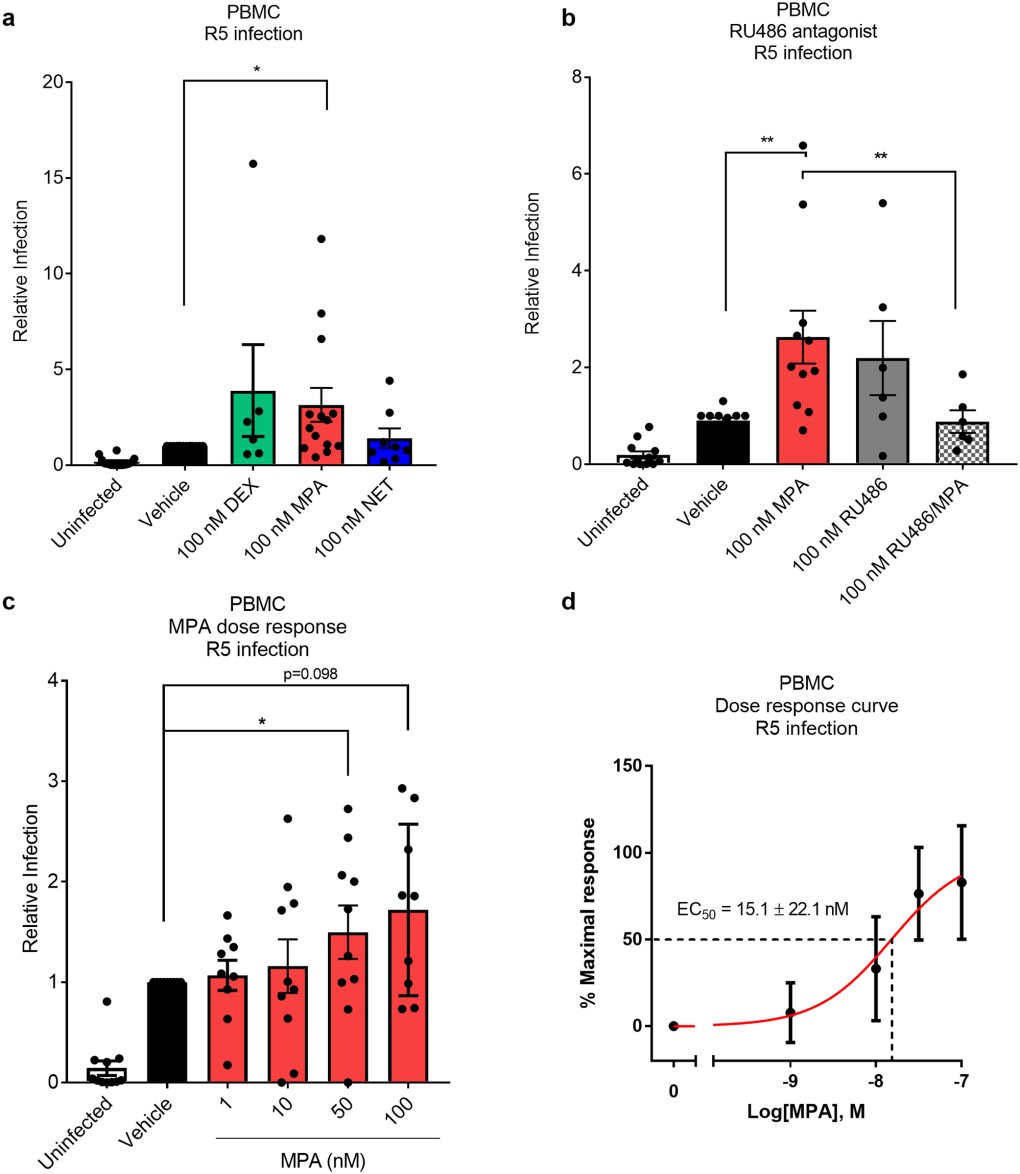
MPA, unlike NET, dose-dependently increases R5 HIV-1 replication in PBMCs in the range of peak serum levels of DMPA users, via a GR-dependent mechanism. (a-d) Non-activated PBMCs were pre-treated with various ligands at the concentrations indicated, or with vehicle control, (0.1% v/v EtOH) for 24 hours. Thereafter, PBMCs were infected with 10 IU/mL HIV-1_BaL-Renilla_ IMC for 2 hours and maintained in full RPMI supplemented with 30 IU/mL IL2 for 5 days before harvesting for *Renilla* luciferase or MTT viability. Relative infection for each donor for progestins relative to vehicle, where the only variable was the absence or presence of progestin, was calculated by normalizing RLU against corresponding MTT values, and calibrated to vehicle set to 100% relative infection. Each condition was performed at least in triplicate. The data (a-d) are represented as mean ± SEM. In (a-c), individual experimental means are depicted as black dots. Statistical analysis was performed using (a-c) a non-parametric Kruskal-Wallis one-way ANOVA with a subsequent Wilcoxon non-parametric t- test to compare vehicle and MPA or (b) a Mann Whitney test when comparing MPA to RU486/MPA. (d) A non-linear regression line was generated to calculate EC_50_ and statistical analysis was performed using a parametric one-way ANOVA with Tukey multiple comparisons post-test comparing all conditions. Statistical significance is indicated with * or ** denoting p<0.05 or p<0.01, respectively. In a) the dexamethasone (Dex) (a synthetic GR agonist) result is from 6 donors, MPA is from 14 donors and NET is from 8 donors, b shows results for 6 donors and c-d show results for 10 donors, where each point on the histogram shows the result from a separate donor.

The direct effects of MPA and NET on HIV-1 replication were investigated in PBMCs *ex vivo*. We found that 100 nM MPA significantly increased R5 HIV-1 replication in non-activated PBMCs from 14 independent female donors by 3.1 ± 0.9 fold, while equimolar NET had no effect (Fig 1a). No significant effects on HIV-1 replication were observed for pooled results from ten independent female donors for PBMCs activated with PHA before exposure to 100 nM MPA or NET (data not shown). Interestingly, we also observed a variable response in HIV-1 replication with the GR-specific agonist dexamethasone (Dex) (Fig 1a), suggesting that synthetic GR agonists increase HIV-1 replication in some donor samples. We have previously shown that PBMCs from female donors express GR mRNA and protein, but do not express detectable levels of PR, AR, ER or MR mRNA or protein [14]. We report here that RU486 abrogates the MPA-induced increase in R5 HIV-1 replication in PBMCs (Fig 1b), providing evidence for a GR-mediated mechanism for increased HIV-1 replication in PBMCs in response to MPA, given our demonstrated lack of PR expression. Towards investigating the dose-dependence of the MPA effect, Fig 1c shows that at both 1 and 10 nM MPA, approximately half of the donor samples showed an increase in HIV-1 replication while about half did not. This wide inter-donor variability in response was observed in all the PBMC experiments. The average increase in HIV-1 replication with MPA shows a typical steroid receptor sigmoidal dose-response curve with an EC_50_ of about 15 nM (Fig 1d), within the range of peak serum levels of DMPA-IM users.

### MPA increases the ratio of CD4+/CD8+ cells, the frequency of activated and CCR5 expressing T-cells and the density of the CCR5 coreceptor on T-cells

**Fig 2:**
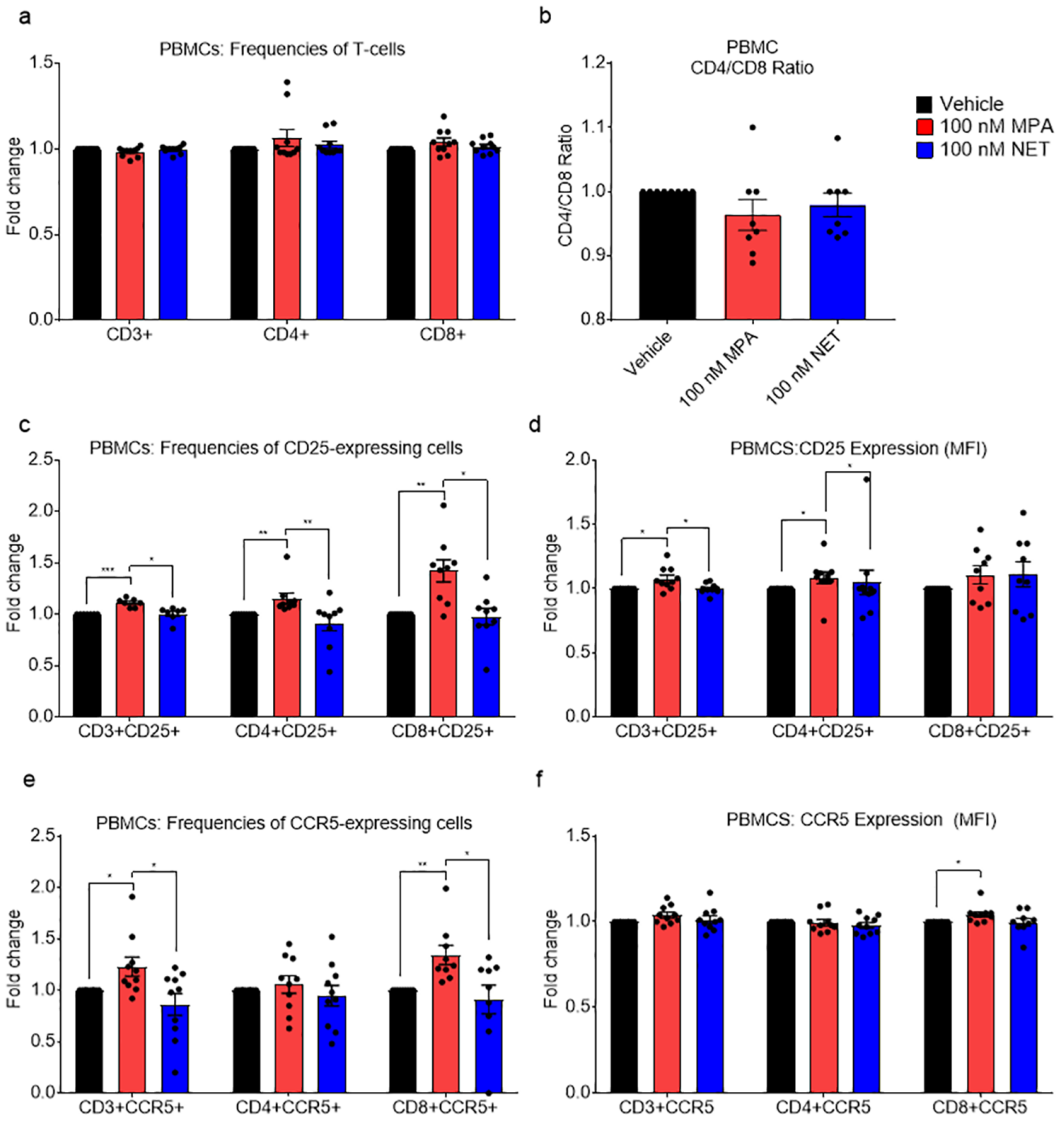
MPA, unlike NET, increases CCR5 levels and activation of T-cells in PBMCs after 24 hours. PBMCs were stimulated with 100 nM MPA, 100 nM NET or vehicle for 24 hours after which relative levels of CD25 and CCR5 were determined in CD3+, CD4+ and CD8+ T-cells using flow cytometry. Results are shown as either relative frequency of CD3+, CD4+ and CD8+ T-cells (a), CD4/CD8 ratios (b), relative frequency of cells expressing CD25 (c) or CCR5 (e), or relative MFI of CD25 (d) or CCR5 (f) in CD3+, CD4+ and CD8+ T-cells. (a, c-f) show results from 10 independent donor experiments, while (b) shows CD4/CD8 ratios for 8 independent donor experiments. The data (a-f) are represented as mean ± SEM. Each point on the histogram shows the result from a separate donor, with experiments for each donor performed with vehicle, MPA and NET in parallel, and the value for vehicle set to 1. Statistical significance was determined by using a non-parametric Kruskal-Wallis one-way ANOVA with Dunn’s post-test with * or ** denoting p<0.05 or p<0.01 respectively.

Towards understanding the mechanisms whereby MPA but not NET affects HIV-1 replication, we investigated whether MPA differentially regulates activation and CCR5 HIV-1 coreceptor levels compared to NET in PBMCs by flow cytometry. The gating strategy is shown in Fig A in S1 File. Exposure of non-activated PBMCs for 24 hours to 100 nM MPA or NET had no significant effects on total cell numbers or frequency of CD3+, CD4+ or CD8+ T-cells (Fig 2a and 2b). Interestingly, 100 nM MPA, but not NET, increased the frequency of activated CD3+, CD4+ and CD8+ T-cells, as measured by CD25 (Fig 2c), as well as the density of CD25 on CD3+ and CD4+ T-cells (Fig 2d). Additionally, we observed a significant increase with 100 nM MPA in the frequency of CD3+CCR5+ and CD8+CCR5+ T-cells, but not CD4+CCR5+ T-cells within the CD3+ population, unlike for 100 nM NET (Fig 2e), with a concomitant increase in CCR5 density in CD8+ T-cells for MPA but not NET (Fig 2f). Tables A and B in S1 File summarize the total percentage of cell types and the average of the MFI densities, respectively, for the above flow cytometry data.

**Fig 3:**
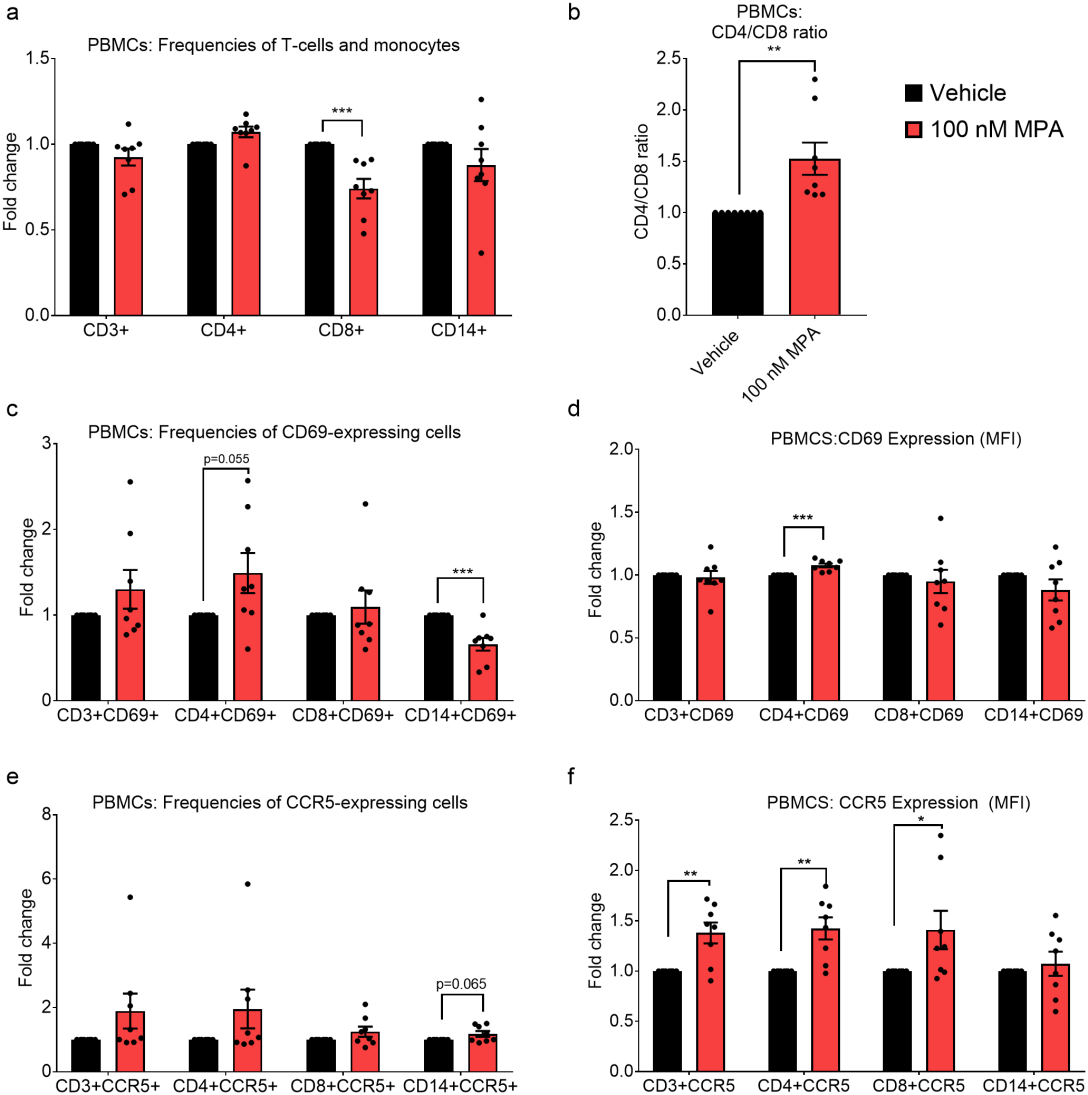
Longer exposure to MPA increases the CD4/CD8 ratio, decreases CD8+ T-cell frequency, and increases the density of the CCR5 coreceptor on CD4+ and CD8+ T-cells. PBMCs were stimulated with 100 nM MPA vehicle for 7 days, after which relative levels of CD69 and CCR5 were determined in CD3+, CD4+ and CD8+ T-cells and CD14+ monocytes using flow cytometry. Results are shown as either relative frequency of CD3+, CD4+ and CD8+ T-cells and CD14+ monocytes (a), CD4/CD8 ratio (b), relative frequency of cells expressing CD69 (c) or CCR5 (e), or relative MFI of CD69 (d) or CCR5 (f) in CD3+, CD4+ and CD8+ T-cells and CD14+ monocytes. Results are shown from 8 independent donor experiments. The data (a-f) are represented as mean ± SEM. Each point on the histograms shows the result from a separate donor, with experiments for each donor performed with vehicle and MPA in parallel, with the values for vehicle set to 1. Data were analyzed using a parametric unpaired t-test comparing vehicle to MPA. Statistical significance is shown with *, ** or *** denoting p<0.05, p<0.01 or p<0.001, respectively.

Having observed that MPA increased the frequency of activated and CCR5-expressing T-cells after 24 hours, we further investigated the effects of time of exposure to MPA by incubating PBMCs with vehicle or 100 nM MPA for 7 days, followed by analysis by flow cytometry. MPA decreased the frequency of CD8+ T-cells (Fig 3a), thereby significantly increasing the CD4/CD8 ratio 1.5-fold (Fig 3b). In addition, MPA increased the frequency and density of CD69 on activated CD4+ cells but decreased the frequency of activated CD69+CD14+ monocytes (Fig 3c and 3d). Furthermore, while having no significant effect on the frequency of CCR5-expressing cells, MPA increased CCR5 MFI or density, in CD3+, CD4+ and CD8+ T-cells (Fig 3e and 3f). Tables C and D in S1 File detail the total percentage of cell types and the average MFI densities, respectively, for the above flow cytometry data.

### MPA, unlike NET, dose-dependently increases HIV-1 replication in a cervical cell line, via the GR

**Fig 4:**
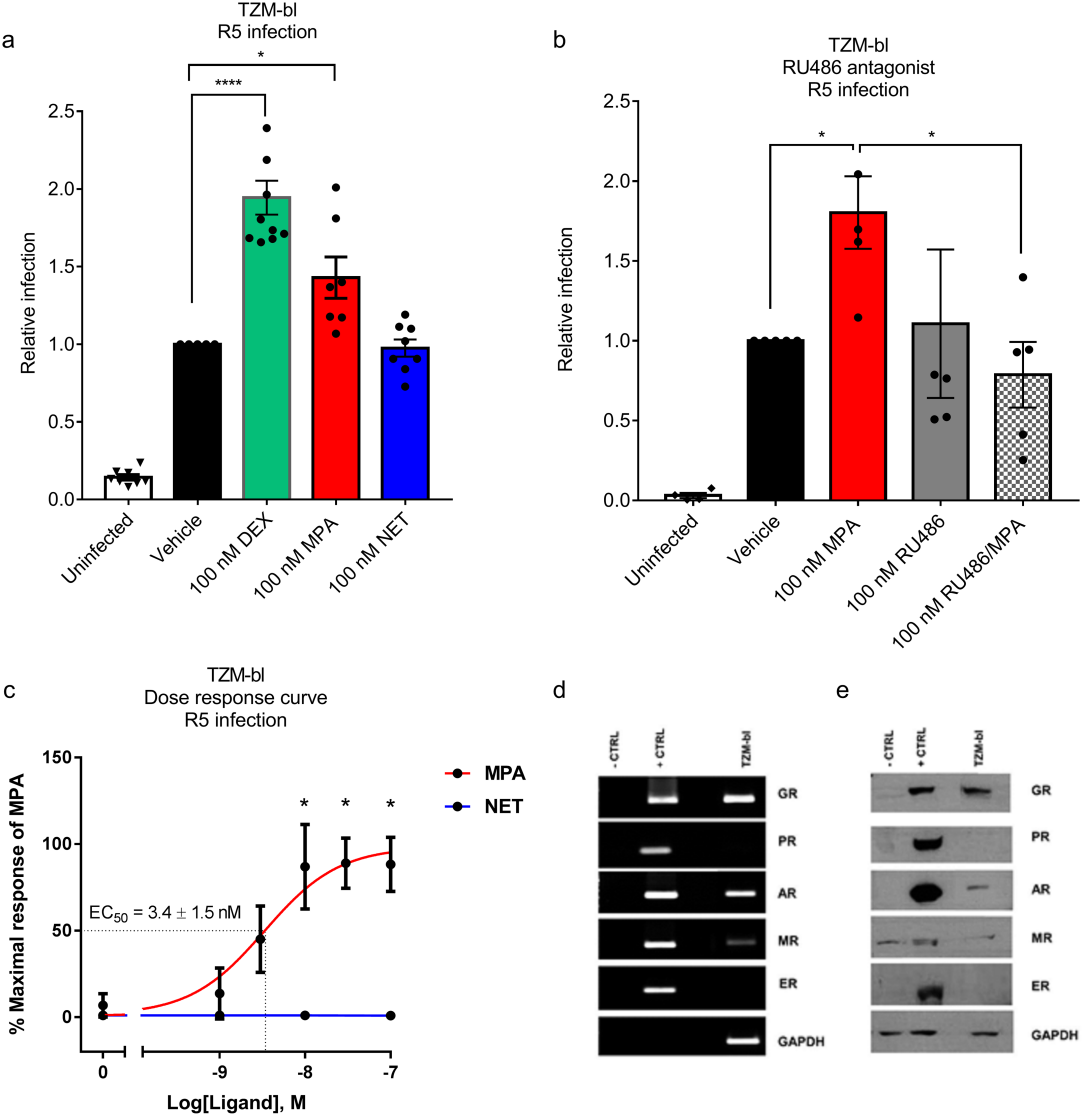
MPA, unlike NET, dose-dependently increases R5 HIV-1 replication in the TZM-bl cervical cell line, via a GR-dependent mechanism. TZM-bl cells were stimulated in parallel for 24 hours with the various ligands, or combinations thereof or vehicle control (0.1% v/v EtOH), at the concentrations indicated, before infection with 20 IU/mL R5 HIV-1_BaL-Renilla_. Samples were harvested 48 hours later for *Renilla* luciferase and MTT viability assays. Each condition was performed at least in triplicate. Relative infection was calculated by normalising RLU against corresponding MTT values. This value for vehicle was set to 1 for (a) and (b), while for (c), data were analysed relative to the maximal response generated by MPA which was set to 100%. Statistical analysis was performed using (a) a parametric one-way ANOVA with Dunnett’s multiple comparisons post test when comparing to vehicle or (b) a non-parametric Kruskal-Wallis one-way ANOVA with a subsequent unpaired t test comparing vehicle and MPA or comparing MPA and MPA/RU486. (c) A non-linear regression line was generated to calculate EC_50_ and statistical analysis was performed using a parametric one-way ANOVA with Tukey multiple comparisons post-test comparing all conditions. Significant differences are shown by *, ** or *** denoting p<0.05, p<0.01 or p<0.001, respectively. The data (a-c) are represented as mean ± SEM and show the results of nine, five and six independent experiments, respectively, with each point performed in triplicate at least. **(d-e) The GR is the predominant steroid receptor protein expressed in the TZM-bl cell line**. Cell lysates were prepared and the steroid receptor mRNA and protein levels were detected by qRT-PCR and western blotting, respectively. (d) indicates that TZM-bl cells express detectable GR, AR and MR mRNA while (e) shows that TZM-bl cells express detectable GR and AR protein.

**Fig 5:**
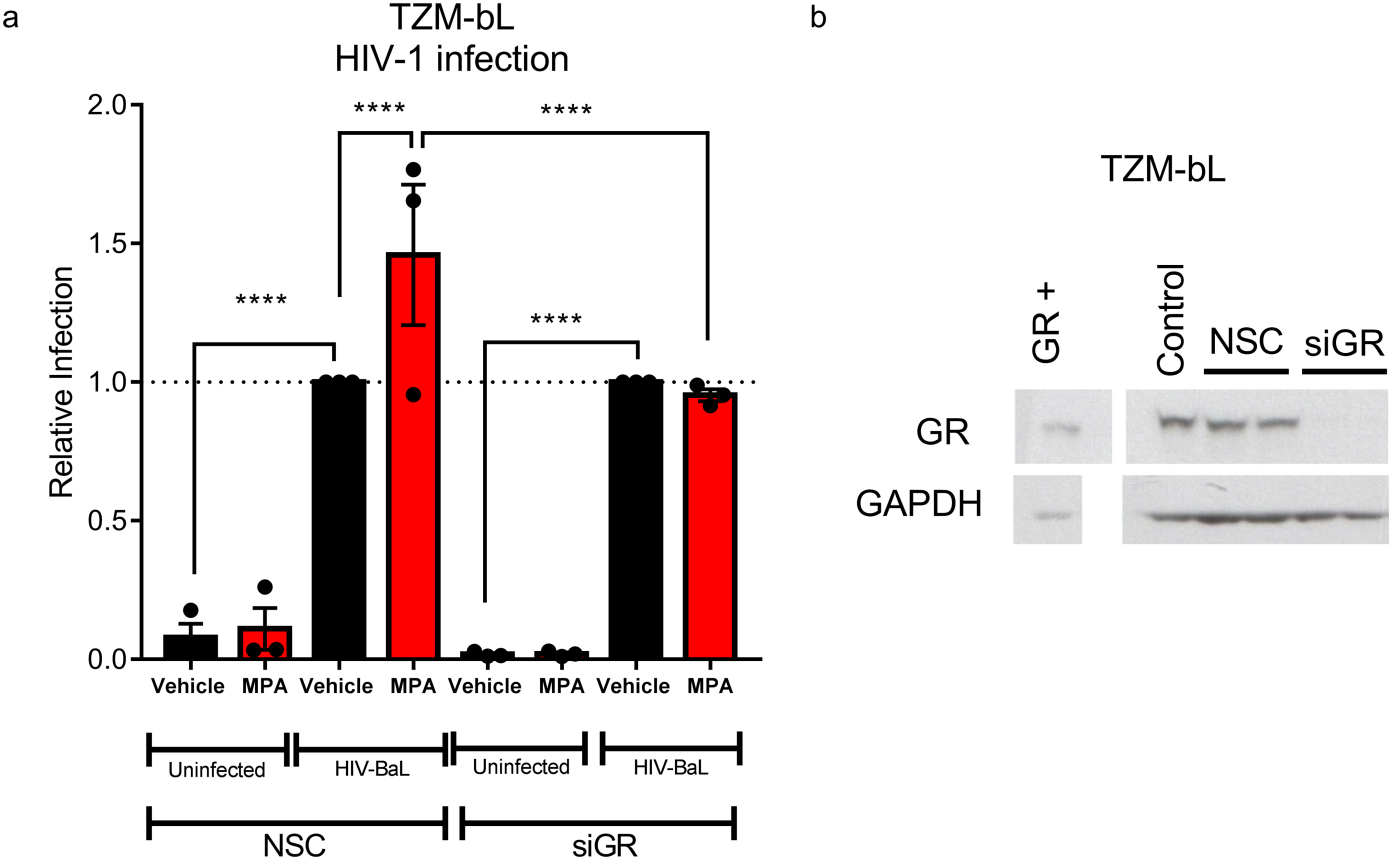
MPA-induced HIV-1 replication requires the GR in TZM-bl cells. TZM-bl cells transfected with 10 nM non-silencing control (NSC) or siRNA targeting the human GR (siGR) for 48 hours. (a) Cells were re-seeded into 96-well plates for 24 hours, followed by stimulation for 24 hours with 100 nM MPA or vehicle (0.1% v/v EtOH), then infected with 10 IU/mL HIV-1_BaL_Renilla_ (“HIV-BaL”) or equivalent volume of virus control (“Uninfected”). Cells were harvested 72 hours later for luciferase (infection) and for MTT (cell viability). The results were pooled from 3 independent experiments where each point was in quadruplicate and are represented as mean ± SEM. Relative infection was calculated as luciferase (RLU) divided by average absorbance at 595nm (MTT) for the quadruplicates. Infection was plotted relative to HIV-1_BaL_Renilla_ vehicle control set to 1. Statistical comparisons were carried out using a two-way ANOVA with Tukey’s multiple comparisons post-test, with **** denoting p<0.001. (b) Cells seeded and transfected in parallel were harvested 48 hours after transfection in SDS sample buffer. Lysates were analyzed for GR levels by western blotting using GAPDH as a loading control. A representative western blot is shown.

To further investigate the mechanism of CCR5 regulation by MPA, we determined whether the effects on HIV-1 replication and CCR5 expression levels could be mimicked in an infectable cell line model. We found that 100 nM of the GR-specific agonist Dex and 100 nM MPA significantly increased R5 HIV-1 replication by 1.9 ± 0.1 and 1.4 ± 0.1 fold, respectively, in cervical TZM-bl cells, unlike equimolar NET (Fig 4a), similar to the effects observed in PBMCs (Fig 1). The MPA-induced effect was completely abrogated with the GR/PR antagonist RU486 (Fig 4b). Dose-response analysis revealed that MPA had a potency or EC_50_ of 3.4 ± 1.5 nM for HIV-1 infection (Fig 4c). TZM-bl cells express both GR and AR mRNA (Fig 4d) and protein (Fig 4e), but do not express detectable PR and ER mRNA (Fig 4d) or protein (Fig 4e). Consistent with this result, we further show that siRNA-mediated knock down of the GR in TZM-bl cells abrogated the MPA-induced increase in HIV-1 replication (Fig 5a). Taken together these results show that MPA-induced increased HIV-1 replication in TZM-bl cells is dependent on the presence of the GR, consistent with a lack of such an effect for NET which does not act via the GR.

### MPA, unlike NET, increases CCR5 levels in TZM-bl cells via a GR-dependent mechanism

**Fig 6:**
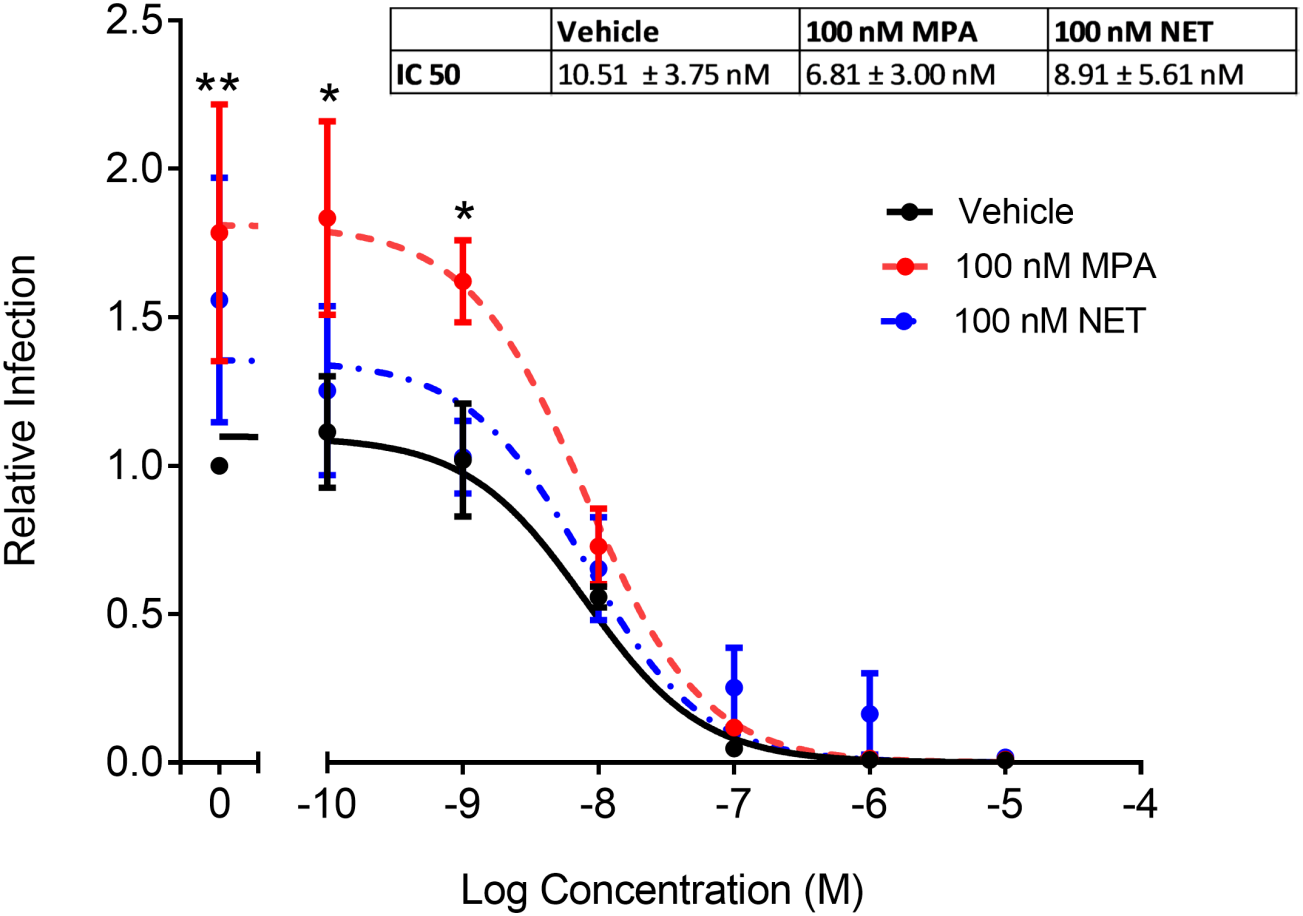
Increased R5 HIV-1 replication with MPA in TZM-bl cells requires the CCR5 coreceptor. TZM-bl cells were treated for 24 hours with 100 nM MPA, 100 nM NET or vehicle control (EtOH, 0.1% v/v). Cells were infected with 20 IU/mL of HIV-1_BaL-Renilla_ or control in the presence of progestins in the absence or presence of varying concentrations of MVC or DMSO. Cells were harvested 48 hours later and infection determined with BrightGlo luciferase. Cell viability was measured by MTT assay and read at an absorbance of 595 nm. Luciferase readings were expressed over MTT and infection determined relative to HIV-1_BaL-Renilla_ vehicle control (EtOH/DMSO, 0.1% v/v) set to 1. Results show MVC dose- response curves for four independent experiments, each performed in triplicate and is represented as mean ± SEM. Non-linear regression was used and generated best fit slopes with R^2^ values of 0.87, 0.81 and 0.63 for vehicle, 100 nM MPA or NET, respectively, to determine IC_50_ of MVC. Statistical significance was assessed using a two-way ANOVA with a post hoc Tukey tests between MPA and NET to the vehicle control, or MPA compared to NET with *, ** denoting p<0.05, p<0.001 respectively.

**Fig 7:**
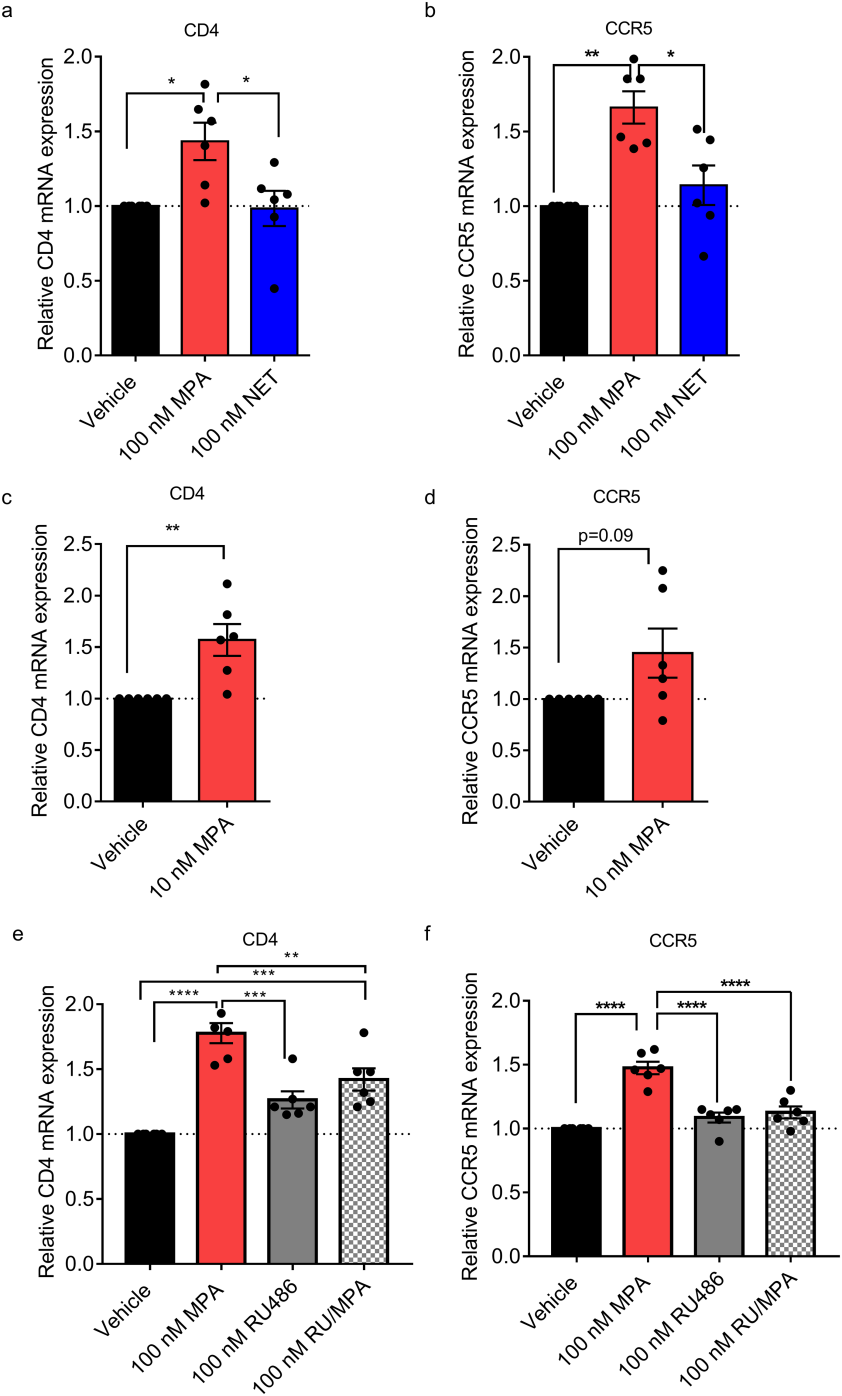
MPA, unlike NET, increases CD4 and CCR5 mRNA levels in TZM-bls, via a GR-dependent mechanism. TZM-bl cells were stimulated for 24 hours with indicated ligands (a-b) 100 nM MPA, 100 nM NET or vehicle control (EtOH) (0.1% v/v ethanol) or (c-d) 10 nM MPA or (e-f) 100 nM MPA, 100 nM RU486 (RU), or combinations thereof (RU/MPA). RNA was isolated, cDNA was synthesised and relative CCR5, CD4 mRNA levels were determined by real time qPCR, normalized to GAPDH and relative fold change in expression was determined by setting vehicle control to 1. Results are shown from 6 independent experiments for each panel and is represented as mean ± SEM. Statistical significance was determined by using (a-b, e-f) one-way ANOVA with a post hoc Tukey tests between conditions or (c-d) using a parametric unpaired t-test comparing the vehicle to 10 nM MPA with * or ** denoting p<0.05 or p<0.01 respectively.

Having shown that MPA but not NET increased R5 HIV-1 replication in PBMCs and TZM-bl cells, we next investigated whether this increase in HIV-1 replication required the CCR5 coreceptor in TZM-bl cells. Experiments with the CCR5 antagonist, maraviroc, revealed that R5 HIV-1 replication in TZM-bl cells requires entry via the CCR5 coreceptor (Fig 6). Interestingly, MPA did not change the potency (EC_50_) or efficacy (maximal response) of maraviroc (Fig 6). We next sought to determine whether the differential increase by MPA compared to NET on HIV-1 replication was due to the differential effects of these progestins on CD4 receptor or CCR5 HIV-1 coreceptor expression levels. Both CD4 and CCR5 mRNA levels were significantly upregulated by 1.6 ± 0.10 (p = 0.007) and 1.4 ± 0.12 (p = 0.019) fold, respectively, by 100 nM MPA but not by equimolar NET, after 24 hours incubation in TZM-bl cells (Fig 7a and 7b). Similar effects were observed with 10 nM MPA (Fig 7c and 7d). No significant differences in CXCR4 coreceptor expression levels were observed with 10 or 100 nM MPA or with 100 nM NET (data not shown). The MPA effect on both CD4 (Fig 7e) and CCR5 (Fig 7f) expression was significantly inhibited by co-incubation with RU486, consistent with a GR-mediated mechanism, although to a lesser extent for CD4 compared to CCR5.

## Discussion

We show for the first time that physiologically-relevant MPA concentrations, unlike equimolar NET, increases HIV-1 replication in non-activated PBMCs from female donors, and HIV-1 infection in the TZM-bl indicator cell line. We observed a dose-dependent increase in HIV-1 replication with MPA, with an EC_50_ of 15.1 ± 22.1 nM in non-activated PBMCs and 3.4 ± 1.5 nM in TZM-bl cells. Reasons for the increased sensitivity to MPA of the cell line compared to PBMCs are not clear but may be due to inherent differences or mechanisms used in PBMCs and TZM-bl cells and could reflect higher HIV-1 receptor, HIV-1 coreceptor or GR levels, or other differences in intracellular mediators [8, 36]. Since serum concentrations of DMPA-IM range from 3–100 nM within the first 20 days post-injection and plateau at about 2.6 nM for 2–3 months after injection [8], our results suggest that these direct effects of MPA on PBMCs occur at physiologically-relevant concentrations as found in DMPA-IM users. Recent clinical data showing increased *ex vivo* infection of CD4+ T-cells in PBMCs from women on DMPA-IM [9] are in support of our findings and suggest that our *ex vivo* findings may be of clinical relevance. Our results are consistent with another report showing that MPA increases HIV-1 pseudovirus infection in non-activated PBMCs at about 1–12.5 nM [17]. Interestingly, another report showed that MPA, but not progesterone, increases R5 HIV-1 IMC replication in activated PBMCs at 1 μM, but not with 10 nM [16]. While we did observe increased HIV-1 replication in some PHA-activated PBMCs at 100 nM MPA, this effect was not significant when results for several donors were pooled (data not shown). Bearing in mind the limitations in directly translating *ex vivo* results to *in vivo*, the high degree of biological variability between donor samples in HIV-1 replication in response to MPA shown in Fig 1 could conceivably translate into variable donor cell responses *in vivo*. Additionally, the finding that 1–100 nM MPA falls in the steep and not the shallower parts of the dose-response curve [8] in Fig 1d further suggests that if these effects occur *in vivo*, women could possibly be particularly prone to differential responses to MPA depending on their MPA serum concentrations in the 1–100 nM range. The possible risks associated with MPA usage may thus depend critically on individual factors such as number of injections, time after injection, metabolism, body weight, BMI, lactation and timing of sexual intercourse after injection. The effects on HIV-1 replication in PBMCs and T-cells may be less significant for DMPA-SC users [7], with similar plateau but lower reported peak serum concentrations (1.6–4.4 nM) [37, 38] than DMPA-IM users, corresponding to a shallower part of the dose-response curve (Fig 1d). In contrast to MPA, our findings suggest that NET-EN is unlikely to affect HIV-1 replication even at peak serum NET concentrations (10–50 nM) [39, 40].

Flow cytometry of PBMCs exposed in parallel to vehicle or equimolar MPA or NET revealed for the first time that MPA, unlike NET, is likely to increase HIV-1 replication via several complimentary mechanisms involving target T-cells. These include increased activation of T-cells, increased ratio of CD4+/CD8+ T-cells and increased CCR5 chemokine receptor expression levels, in a manner dependent on time of exposure to MPA. Specifically, 24 hour stimulation with MPA, unlike NET, increased the frequency of CD3+, CD4+ and CD8+ T-cells expressing the early activation marker, CD25. Longer (7-day) stimulation with MPA increased expression of the activation marker CD69 in CD4+ T-cells, with a near-significant increase (p = 0.055) in CD69+CD4+ T cell frequency, indicating increased activation of CD4+ T-cells. This increase in T-cell activation by MPA would be consistent with increased T-cell infection as previously reported [41–43]. The CD4/CD8 ratio of greater than 1 found in this study is consistent with the number of CD4 outnumbering CD8 cells in PBMCS, as previously reported [44, 45]. The CD4/CD8 ratio increased approximately 1.6-fold from day 2 to day 7 indicating a general increase after 7 days of culture (Tables A and C in S1 File). More importantly though long-term MPA stimulation (7 days) significantly increased the CD4/CD8 ratio further in PBMCs. This, together with the increase in frequency of activated CD4+ cells and significant decrease in % CD8+ cells after 7 days, suggests that MPA promotes infection by increasing the frequency of infectable cells while concomitantly decreasing the frequency of cytotoxic cells.

When comparing our flow cytometry data to those in the literature, limited data exist on the clinical effects of DMPA-IM (or predominant DMPA-IM usage) on PBMCs in women [9, 44, 46], while there is no data for DMPA-SC or NET-EN, and limited data for other forms of hormonal contraception [46]. A recent paper showed that PBMCs from women on DMPA-IM exhibit altered immune markers and increased *ex vivo* infection with R5 HIV-1 [9], consistent with our MPA results. Interestingly they observed increased activation (CD38 marker) for CD4+ T-cells in PBMCs from women on DMPA-IM 1 month after injection and a significant difference at 3 months compared to 1 month after injection [9]. This broad consistency with our results suggests that effects of DMPA-IM on PBMCs *in vivo* are likely due to direct actions of MPA on PBMCs and are dose-dependent, since MPA serum levels are higher 1 month (C_max_ average about 10–20 nM) compared to 3 months (C_min_ 1–3 nM) after injection [7, 8]. These authors did not investigate changes in CD8+ T-cell frequency [9]. Others have found that while the CD4/CD8 ratio was not affected in PBMCs from women using combined oral contraceptives [47], it increased in DMPA-IM users [48], consistent with our *ex vivo* results. In contrast, the increased CD4/CD8 ratio observed by others in postmenopausal NET users [49] may be particular to the postmenopausal state. There are limited flow cytometry data investigating the direct *ex vivo* effects of MPA in PBMCs [17, 50] and none for NET. One report observed no effect with up to 8 nM MPA on the frequency of activated CD4+ T-cells or density of activation markers, as assessed by CD69, CD25 or HLA-DR markers [17]. Similarly, MPA had no effect on dendritic cell activation as assessed by HLA-DR expression [50]. These data may differ from our findings due to the shorter period of incubation with MPA (24 or 48 hours) or other methodological differences.

Changes in frequency of CCR5-expressing cells and density of CCR5 chemokine receptor expression is another mechanism whereby R5 HIV-1 infection and replication could be increased in target T-cells. Decreased expression of CCR5 in PBMCs has been shown to correlate with reduced HIV-1 infectivity *in vitro* [18]. We show for the first time that in PBMCs, MPA unlike NET increased the frequency of CCR5-expressing CD3+ and CD8+ T-cells after 24 hours, and MPA increased CCR5 expression on CD3+, CD4+ and CD8+ T-cells after 7 days. The recent paper from Tasker et al. showed an increasing trend for both activation and CCR5 density for CD4+ T-cells in PBMCs from women on DMPA-IM [9], consistent with our MPA results. They also showed that *ex vivo* R-tropic HIV-1 infected CD4+ cells in PBMCs from women on DMPA-IM with have a higher CCR5 density than uninfected PBMCs [9], also consistent with the argument that increased CCR5 density increases R-tropic HIV infection of T-cells. Our results are also consistent with previous data in PBMCs from women on DMPA-IM in which CCR5 expression increased in CD4+ and CD8+ T-cells, although there was no reported increase in the frequency of CCR5-expressing CD4+ or CD8+ T-cells [46]. However, others have shown an increase in CCR5 expression and frequency of T-cells expressing CCR5 in cervical but not matched PBMCs in women predominantly using DMPA-IM [44]. Interestingly, others have shown an increased cell surface density of CCR5 on both CD4+ and CD8+ T lymphocyte subsets in PBMCs from women on combined oral contraceptives, compared to no hormone [47], suggesting that this effect may not be unique to MPA. Limited *ex vivo* PBMC data include one report that reported 1 μM, but not 100 nM, MPA prevented the down-regulation of CXCR4 and CCR5 HIV-1 coreceptors on the surface of T-cells after activation and increased HIV-1 replication in activated PBMCs [16]. The latter results do not exclude the possibility that MPA increases CCR5 expression and activation of non-activated PBMCs. Our results do not exclude the possibility that MPA also increases the frequency and expression of the integrin α4β7 on CD4+ T-cells in PBMCs as recently reported by Tasker et al. [9], a topic for future investigation.

Since MPA but not NET increases HIV-1 replication in TZM-bl cells as for PBMCs, we investigated this apparently conserved mechanism further in this cell line model. We showed using the CCR5 antagonist maraviroc that R5 HIV-1 replication requires entry via the CCR5 coreceptor in TZM-bl cells. Using the GR/PR antagonist, RU486, we found that MPA-associated infection was inhibited in both PBMCs and TZM-bl cells. Since PR is not detectable in PBMCs or TZM-bl cells, it is likely that the GR is mediating the response in both these models. This was confirmed by knockdown of the GR using siRNA, which significantly inhibited the MPA effect on HIV-1 infection in TZM-bl cells. Our data provide evidence for the first time that the mechanism via which MPA, unlike NET, increases R5 HIV-1 replication in both PBMCs and TZM-bl cells is mediated via the GR. Attempts to explore these mechanisms in T-cell lines were not successful since the GR was not active in these cells (data not shown). Fortuitously, the use of the TZM-bl cell line contributed additional novel insight, since they do not undergo activation as observed in T-cells, and would presumably lack many of the receptors and signalling pathways found in T-cells. The finding that MPA but not NET increased expression of both the CD4 receptor and the CCR5 coreceptor but not the CXCR4 coreceptor in TZM-bl cells suggests that MPA, unlike NET, upregulates CCR5 expression independently of T-cell activation or other T cell-specific signalling pathways. Changes to CD4/CCR5 ratios are also an important factor controlling HIV infection both in TZM-bl cells and PBMCs [36, 51]. Our TZM-bl results further support a role for GR in mediating MPA-induced infection via changes in CD4 and CCR5 levels, since RU486 significantly inhibited the MPA-induced increase in CCR5 and CD4 mRNA levels. The % inhibition by RU486 was less for CD4 than CCR5, suggesting that other mechanisms may be involved in the MPA regulation of CD4 mRNA. The intracellular mechanism whereby MPA but not NET increases CCR5 mRNA levels in TZM-bl cells is intriguing since these cells are engineered from HeLa cells to express exogenous CD4, CCR5 and CXCR4 [51]. However, we (data not shown) and others have shown that HeLa cells do also express these receptors endogenously [52]. Further detailed investigations on this mechanism are ongoing and beyond the scope of this study.

Taken together, our data strongly support a biological mechanism for increased HIV-1 replication in T-cells based on intrinsic differences between the actions of MPA compared to NET, via GR-mediated mechanisms, which discriminates between MPA and NET. Supporting this are our previous findings that MPA and NET exert differential effects on expression of select genes via the GR in several model systems [10–13]. Our PBMC and TZM-bl cell results are consistent with a predominant mechanism involving MPA-induced increased CD4 and CCR5 expression on T-cells, unlike for NET. It would be interesting to further investigate whether MPA increases CD4+ T-cell proliferation as a possible additional mechanism. The findings in the present study are consistent with tissue explant data (unpublished) adding support for a GR-mediated mechanism involving an MPA-induced increase in CD4 and CCR5 expression in target T-cells. Our data collectively suggest that MPA unlike NET, may increase activation of target systemic and genital tract T-cells, thus supporting higher degrees of viral replication than resting cells and likely acceleration of viral dissemination by T-cells after exposure to HIV in the female genital tract. Our results showing an increased HIV-1 replication in PBMCs may also have physiological implications for effects of MPA on disease progression. Although limited studies to date suggest that DMPA does not have significant effects on AIDS disease progression [53–55], these may occur in some individuals but may not be apparent in a large population analysis and may also depend on time of DMPA usage and time after injection. Additionally, our previous [14] results suggest that MPA, but not NET, may have significant effects on T-cell apoptosis in some women at peak serum concentrations, which may depend critically on weight, metabolism and immune status. Our dose-response results also suggest that individual patient responses to MPA may depend critically on dose, time after injection and intrinsic factors that affect serum concentrations in women, perhaps explaining the variable clinical results to date on biomarkers and infection. However, further investigations are required to determine whether the biological mechanisms elucidated in our *ex vivo* experiments, also occur *in vivo*.

## Supporting information

S1 File Fig A. Gating strategy employed in this study. Table A. Frequencies of PBMC leukocytes and cells expressing CD25 or CCR5 in PBMCs stimulated with 100 nM MPA versus control for 24 hours. Table B. Density of CD25 or CCR5 in PBMC leukocytes stimulated with 100 nM MPA versus control for 24 hours. Table C. Frequencies of PBMC leukocytes and cells expressing CD69 or CCR5 in PBMCs stimulated with 100 nM MPA versus control for 7 days. Table D. Density of CD69 or CCR5 in PBMC leukocytes stimulated with 100 nM MPA versus control for 7 days.
(DOCX)

## References

[1] Polis CB, Curtis KM, Hannaford PC, Phillips SJ, Chipato T, Kiarie JN, et al An updated systematic review of epidemiological evidence on hormonal contraceptive methods and HIV acquisition in women. AIDS. 2016;30(17):2665–83. doi: 10.1097/QAD.0000000000001228 27500670 10.1097/QAD.0000000000001228 PMC5106090

[2] Morrison CS, Chen PL, Kwok C, Baeten JM, Brown J, Crook AM, et al Hormonal contraception and the risk of HIV acquisition: an individual participant data meta-analysis. PLoS Med. 2015;12(1):e1001778 doi: 10.1371/journal.pmed.1001778 25612136 10.1371/journal.pmed.1001778 PMC4303292

[3] Ralph LJ, Gollub EL, Jones HE. Hormonal contraceptive use and women’s risk of HIV acquisition: priorities emerging from recent data. Curr Opin Obstet Gynecol. 2015;27(6):487–95. doi: 10.1097/GCO.0000000000000228 26536211 10.1097/GCO.0000000000000228

[4] Butler AR, Smith JA, Polis CB, Gregson S, Stanton D, Hallett TB. Modelling the global competing risks of a potential interaction between injectable hormonal contraception and HIV risk. AIDS. 2013;27(1):105–13. doi: 10.1097/QAD.0b013e32835a5a52 23014519 10.1097/QAD.0b013e32835a5a52 PMC4862571

[5] ECHO. The Evidence for Contraceptive Options and HIV Outcomes (ECHO) Study 2016. http://echo-consortium.com/.

[6] World Health Organisation. Hormonal contraceptive eligibility for women at high risk of HIV. Guidance Statement: Recommendations concerning the use of hormonal contraceptive methods by women at high risk of HIV. Geneva, Switzerland: World Health Organisation; 2017. http://apps.who.int/iris/bitstream/10665/254662/1/WHO-RHR-17.04-eng.pdf.

[7] Polis CB, Achilles SL, Hel Z, Hapgood JP. Is a lower-dose, subcutaneous contraceptive injectable containing depot medroxyprogesterone acetate likely to impact women’s risk of HIV? Contraception. 2017. Forthcoming. 10.1016/j.contraception.2017.12.003 PMC5828886 29242082

[8] Hapgood JP, Kaushic C, Hel Z. Hormonal Contraception and HIV-1 Acquisition: Biological Mechanisms. Endocr Rev. 2017. Forthcoming. 10.1210/er.2017-00103 PMC5807094 29309550

[9] Tasker C, Davidow A, Roche NE, Chang TL. Depot medroxyprogesterone acetate administration alters immune markers for HIV preference and increases susceptibility of peripheral CD4(+) T-cells to HIV infection. Immunohorizons. 2017;1(9):223–35. doi: 10.4049/immunohorizons.1700047 29188238 10.4049/immunohorizons.1700047 PMC5703073

[10] Ronacher K, Hadley K, Avenant C, Stubsrud E, Simons SS Jr, Louw A, et al Ligand-selective transactivation and transrepression via the glucocorticoid receptor: role of cofactor interaction. Mol Cell Endocrinol. 2009;299(2):219–31. doi: 10.1016/j.mce.2008.10.008 19007848 10.1016/j.mce.2008.10.008

[11] Koubovec D, Ronacher K, Stubsrud E, Louw A, Hapgood JP. Synthetic progestins used in HRT have different glucocorticoid agonist properties. Mol Cell Endocrinol. 2005;242(1–2):23–32. doi: 10.1016/j.mce.2005.07.001 16125839 10.1016/j.mce.2005.07.001

[12] Govender Y, Avenant C, Verhoog NJ, Ray RM, Grantham NJ, Africander D, et al The injectable-only contraceptive medroxyprogesterone acetate, unlike norethisterone acetate and progesterone, regulates inflammatory genes in endocervical cells via the glucocorticoid receptor. PLoS One. 2014;9(5):e96497 doi: 10.1371/journal.pone.0096497 24840644 10.1371/journal.pone.0096497 PMC4026143

[13] Hapgood JP, Ray RM, Govender Y, Avenant C, Tomasicchio M. Differential glucocorticoid receptor-mediated effects on immunomodulatory gene expression by progestin contraceptives: implications for HIV-1 pathogenesis. Am J Reprod Immunol. 2014;71(6):505–12. doi: 10.1111/aji.12214 24547700 10.1111/aji.12214

[14] Tomasicchio M, Avenant C, Du Toit A, Ray RM, Hapgood JP. The progestin-only contraceptive medroxyprogesterone acetate, but not norethisterone acetate, enhances HIV-1 Vpr-mediated apoptosis in human CD4+ T cells through the glucocorticoid receptor. PLoS One. 2013;8(5):e62895 doi: 10.1371/journal.pone.0062895 23658782 10.1371/journal.pone.0062895 PMC3643923

[15] Huijbregts RP, Michel KG, Hel Z. Effect of progestins on immunity: medroxyprogesterone but not norethisterone or levonorgestrel suppresses the function of T cells and pDCs. Contraception. 2014;90(2):123–9. doi: 10.1016/j.contraception.2014.02.006 24674041 10.1016/j.contraception.2014.02.006 PMC4874781

[16] Huijbregts RP, Helton ES, Michel KG, Sabbaj S, Richter HE, Goepfert PA, et al Hormonal contraception and HIV-1 infection: medroxyprogesterone acetate suppresses innate and adaptive immune mechanisms. Endocrinology. 2013;154(3):1282–95. doi: 10.1210/en.2012-1850 23354099 10.1210/en.2012-1850 PMC3578997

[17] Sampah ME, Laird GM, Blankson JN, Siliciano RF, Coleman JS. Medroxyprogesterone acetate increases HIV-1 infection of unstimulated peripheral blood mononuclear cells in vitro. AIDS. 2015;29(10):1137–46. doi: 10.1097/QAD.0000000000000681 26035316 10.1097/QAD.0000000000000681 PMC4453018

[18] Vassiliadou N, Tucker L, Anderson DJ. Progesterone-induced inhibition of chemokine receptor expression on peripheral blood mononuclear cells correlates with reduced HIV-1 infectability in vitro. J Immunol. 1999;162(12):7510–8. 10358206

[19] Africander D, Verhoog N, Hapgood JP. Molecular mechanisms of steroid receptor-mediated actions by synthetic progestins used in HRT and contraception. Steroids. 2011;76(7):636–52. doi: 10.1016/j.steroids.2011.03.001 21414337 10.1016/j.steroids.2011.03.001

[20] Stanczyk FZ, Hapgood JP, Winer S, Mishell DR Jr. Progestogens used in postmenopausal hormone therapy: differences in their pharmacological properties, intracellular actions, and clinical effects. Endocr Rev. 2013;34(2):171–208. doi: 10.1210/er.2012-1008 23238854 10.1210/er.2012-1008 PMC3610676

[21] Hapgood JP, Koubovec D, Louw A, Africander D. Not all progestins are the same: implications for usage. Trends Pharmacol Sci. 2004;25(11):554–7. doi: 10.1016/j.tips.2004.09.005 15491776 10.1016/j.tips.2004.09.005

[22] Stanczyk FZ. All progestins are not created equal. Steroids. 2003;68(10–13):879–90. 14667980 10.1016/j.steroids.2003.08.003

[23] Africander D, Louw R, Hapgood JP. Investigating the anti-mineralocorticoid properties of synthetic progestins used in hormone therapy. Biochem Biophys Res Commun. 2013;433(3):305–10. doi: 10.1016/j.bbrc.2013.02.086 23473756 10.1016/j.bbrc.2013.02.086

[24] Africander DJ, Storbeck KH, Hapgood JP. A comparative study of the androgenic properties of progesterone and the progestins, medroxyprogesterone acetate (MPA) and norethisterone acetate (NET-A). J Steroid Biochem Mol Biol. 2014;143:404–15. doi: 10.1016/j.jsbmb.2014.05.007 24861265 10.1016/j.jsbmb.2014.05.007

[25] Hapgood JP, Africander D, Louw R, Ray RM, Rohwer JM. Potency of progestogens used in hormonal therapy: toward understanding differential actions. J Steroid Biochem Mol Biol. 2014;142:39–47. doi: 10.1016/j.jsbmb.2013.08.001 23954501 10.1016/j.jsbmb.2013.08.001

[26] Kleynhans L, Du Plessis N, Black GF, Loxton AG, Kidd M, van Helden PD, et al Medroxyprogesterone acetate alters Mycobacterium bovis BCG-induced cytokine production in peripheral blood mononuclear cells of contraceptive users. PLoS One. 2011;6(9):e24639 doi: 10.1371/journal.pone.0024639 21931790 10.1371/journal.pone.0024639 PMC3169620

[27] Koubovec D, Vanden Berghe W, Vermeulen L, Haegeman G, Hapgood JP. Medroxyprogesterone acetate downregulates cytokine gene expression in mouse fibroblast cells. Mol Cell Endocrinol. 2004;221(1–2):75–85. doi: 10.1016/j.mce.2004.03.006 15223134 10.1016/j.mce.2004.03.006

[28] Louw-du Toit R, Hapgood JP, Africander D. Medroxyprogesterone Acetate Differentially Regulates Interleukin (IL)-12 and IL-10 in a Human Ectocervical Epithelial Cell Line in a Glucocorticoid Receptor (GR)-dependent Manner. J Biol Chem. 2014;289(45):31136–49. doi: 10.1074/jbc.M114.587311 25202013 10.1074/jbc.M114.587311 PMC4223317

[29] Edmonds TG, Ding H, Yuan X, Wei Q, Smith KS, Conway JA, et al Replication competent molecular clones of HIV-1 expressing Renilla luciferase facilitate the analysis of antibody inhibition in PBMC. Virology. 2010;408(1):1–13. doi: 10.1016/j.virol.2010.08.028 20863545 10.1016/j.virol.2010.08.028 PMC2993081

[30] Pear WS, Nolan GP, Scott ML, Baltimore D. Production of high-titer helper-free retroviruses by transient transfection. Proc Natl Acad Sci U S A. 1993;90(18):8392–6. 7690960 10.1073/pnas.90.18.8392 PMC47362

[31] Reed LJ, Muench H. A simple method of estimating fifty percent endpoints. Am J Hyg. 1938;27(3):493–7.

[32] Eszterhas SK, Ilonzo NO, Crozier JE, Celaj S, Howell AL. Nanoparticles containing siRNA to silence CD4 and CCR5 reduce expression of these receptors and inhibit HIV-1 infection in human female reproductive tract tissue explants. Infect Dis Rep. 2011;3(2):e11 doi: 10.4081/idr.2011.e11 24470908 10.4081/idr.2011.e11 PMC3892589

[33] Verhoog NJ, Du Toit A, Avenant C, Hapgood JP. Glucocorticoid-independent repression of tumor necrosis factor (TNF) alpha-stimulated interleukin (IL)-6 expression by the glucocorticoid receptor: a potential mechanism for protection against an excessive inflammatory response. J Biol Chem. 2011;286(22):19297–310. doi: 10.1074/jbc.M110.193672 21474440 10.1074/jbc.M110.193672 PMC3103308

[34] Pfaffl MW. A new mathematical model for relative quantification in real-time RT-PCR. Nucleic Acids Res. 2001;29(9):e45 11328886 10.1093/nar/29.9.e45 PMC55695

[35] Avenant C, Kotitschke A, Hapgood JP. Glucocorticoid receptor phosphorylation modulates transcription efficacy through GRIP-1 recruitment. Biochemistry. 2010;49(5):972–85. doi: 10.1021/bi901956s 20047289 10.1021/bi901956s

[36] Polonis VR, Brown BK, Rosa Borges A, Zolla-Pazner S, Dimitrov DS, Zhang MY, et al Recent advances in the characterization of HIV-1 neutralization assays for standardized evaluation of the antibody response to infection and vaccination. Virology. 2008;375(2):315–20. doi: 10.1016/j.virol.2008.02.007 18367229 10.1016/j.virol.2008.02.007

[37] Jain J, Dutton C, Nicosia A, Wajszczuk C, Bode FR, Mishell DR Jr. Pharmacokinetics, ovulation suppression and return to ovulation following a lower dose subcutaneous formulation of Depo-Provera. Contraception. 2004;70(1):11–8. doi: 10.1016/j.contraception.2004.01.011 15208047 10.1016/j.contraception.2004.01.011

[38] Toh YC, Jain J, Rahnny MH, Bode FR, Ross D. Suppression of ovulation by a new subcutaneous depot medroxyprogesterone acetate (104 mg/0.65 mL) contraceptive formulation in Asian women. Clin Ther. 2004;26(11):1845–54. doi: 10.1016/j.clinthera.2004.11.013 15639696 10.1016/j.clinthera.2004.11.013

[39] Fotherby K, Saxena BN, Shrimanker K, Hingorani V, Takker D, Diczfalusy E, et al A preliminary pharmacokinetic and pharmacodynamic evaluation of depot-medroxyprogesterone acetate and norethisterone oenanthate. Fertil Steril. 1980;34(2):131–9. 7409232 10.1016/s0015-0282(16)44895-8

[40] Sang GW, Fotherby K, Howard G, Elder M, Bye PG. Pharmacokinetics of norethisterone oenanthate in humans. Contraception. 1981;24(1):15–27. 7273765 10.1016/0010-7824(81)90065-2

[41] Haase AT. Perils at mucosal front lines for HIV and SIV and their hosts. Nat Rev Immunol. 2005;5(10):783–92 16200081 10.1038/nri1706

[42] Stevenson M, Stanwick TL, Dempsey MP, Lamonica CA. HIV-1 replication is controlled at the level of T cell activation and proviral integration. EMBO J. 1990;9(5):1551–60. 2184033 10.1002/j.1460-2075.1990.tb08274.x PMC551849

[43] Meditz AL, Haas MK, Folkvord JM, Melander K, Young R, McCarter M, et al HLA-DR+ CD38+ CD4+ T lymphocytes have elevated CCR5 expression and produce the majority of R5-tropic HIV-1 RNA in vivo. J Virol. 2011;85(19):10189–200. doi: 10.1128/JVI.02529-10 21813616 10.1128/JVI.02529-10 PMC3196402

[44] Byrne EH, Anahtar MN, Cohen KE, Moodley A, Padavattan N, Ismail N, et al Association between injectable progestin-only contraceptives and HIV acquisition and HIV target cell frequency in the female genital tract in South African women: a prospective cohort study. Lancet Infect Dis. 2016;16(4):441–8. doi: 10.1016/S1473-3099(15)00429-6 26723758 10.1016/S1473-3099(15)00429-6 PMC5294917

[45] Iyer SS, Sabula MJ, Mehta CC, Haddad LB, Brown NL, Amara RR, et al Characteristics of HIV target CD4 T cells collected using different sampling methods from the genital tract of HIV seronegative women. PLoS One. 2017;12(6):e0178193 doi: 10.1371/journal.pone.0178193 28570576 10.1371/journal.pone.0178193 PMC5453484

[46] Sciaranghella G, Wang C, Hu H, Anastos K, Merhi Z, Nowicki M, et al CCR5 Expression Levels in HIV-Uninfected Women Receiving Hormonal Contraception. J Infect Dis. 2015;212(9):1397–401. doi: 10.1093/infdis/jiv233 25895986 10.1093/infdis/jiv233 PMC4601918

[47] Prakash M, Kapembwa MS, Gotch F, Patterson S. Oral contraceptive use induces upregulation of the CCR5 chemokine receptor on CD4(+) T cells in the cervical epithelium of healthy women. J Reprod Immunol. 2002;54(1–2):117–31. 11839399 10.1016/s0165-0378(01)00125-5

[48] Sridama V, Limpongsanurak S, Sritippayawan S, Youngprapakorn S. Decreased suppressor T-lymphocytes in women who received progestogen injections. J Med Assoc Thai. 1992;75(8):479–82. 1300365

[49] Dogan E, Erkoc R, Demir C, Sayarlioglu H, Dilek I, Sayarlioglu M. Effect of hormone replacement therapy on CD4+ and CD8+ numbers, CD4+/CD8+ ratio, and immunoglobulin levels in hemodialysis patients. Ren Fail. 2005;27(4):421–4. 16060130

[50] Quispe Calla NE, Ghonime MG, Cherpes TL, Vicetti Miguel RD. Medroxyprogesterone acetate impairs human dendritic cell activation and function. Hum Reprod. 2015;30(5):1169–77. doi: 10.1093/humrep/dev035 25740884 10.1093/humrep/dev035 PMC4481667

[51] Platt EJ, Wehrly K, Kuhmann SE, Chesebro B, Kabat D. Effects of CCR5 and CD4 cell surface concentrations on infections by macrophagetropic isolates of human immunodeficiency virus type 1. J Virol. 1998;72(4):2855–64. 9525605 10.1128/jvi.72.4.2855-2864.1998 PMC109730

[52] Sales KJ, Adefuye A, Nicholson L, Katz AA. CCR5 expression is elevated in cervical cancer cells and is up-regulated by seminal plasma. Mol Hum Reprod. 2014;20(11):1144–57. doi: 10.1093/molehr/gau063 25103627 10.1093/molehr/gau063

[53] Phillips SJ, Curtis KM, Polis CB. Effect of hormonal contraceptive methods on HIV disease progression: a systematic review. AIDS. 2013;27(5):787–94. doi: 10.1097/QAD.0b013e32835bb672 23135169 10.1097/QAD.0b013e32835bb672

[54] Phillips SJ, Polis CB, Curtis KM. The safety of hormonal contraceptives for women living with HIV and their sexual partners. Contraception. 2016;93(1):11–6. doi: 10.1016/j.contraception.2015.10.002 26515194 10.1016/j.contraception.2015.10.002

[55] Heffron R, Mugo N, Ngure K, Celum C, Donnell D, Were E, et al Hormonal contraceptive use and risk of HIV-1 disease progression. AIDS. 2013;27(2):261–7. doi: 10.1097/QAD.0b013e32835ad473 23079806 10.1097/QAD.0b013e32835ad473 PMC3740957

