# Supplementary material for: Medroxyprogesterone acetate, unlike norethisterone, increases HIV-1 replication in human peripheral blood mononuclear cells and an indicator cell line, via mechanisms involving the glucocorticoid receptor, increased CD4/CD8 ratios and CCR5 levels
Source: PLoS One. 2018 Apr 26;13(4):e0196043. doi: 10.1371/journal.pone.0196043 (PMC5919616; doi:10.1371/journal.pone.0196043)
Supplement: S1 File — Fig A. Gating strategy employed in this study. Table A. Frequencies of PBMC leukocytes and cells expressing CD25 or CCR5 in PBMCs stimulated with 100 nM MPA versus control for 24 hours. Table B. Density of CD25 or CCR5 in PBMC leukocytes stimulated with 100 nM MPA versus control for 24 hours. Table C. Frequencies of PBMC leukocytes and cells expressing CD69 or CCR5 in PBMCs stimulated with 100 nM MPA versus control for 7 days. Table D. Density of CD69 or CCR5 in PBMC leukocytes stimulated with 100 nM MPA versus control for 7 days. (DOCX) [file pone.0196043.s001.docx]

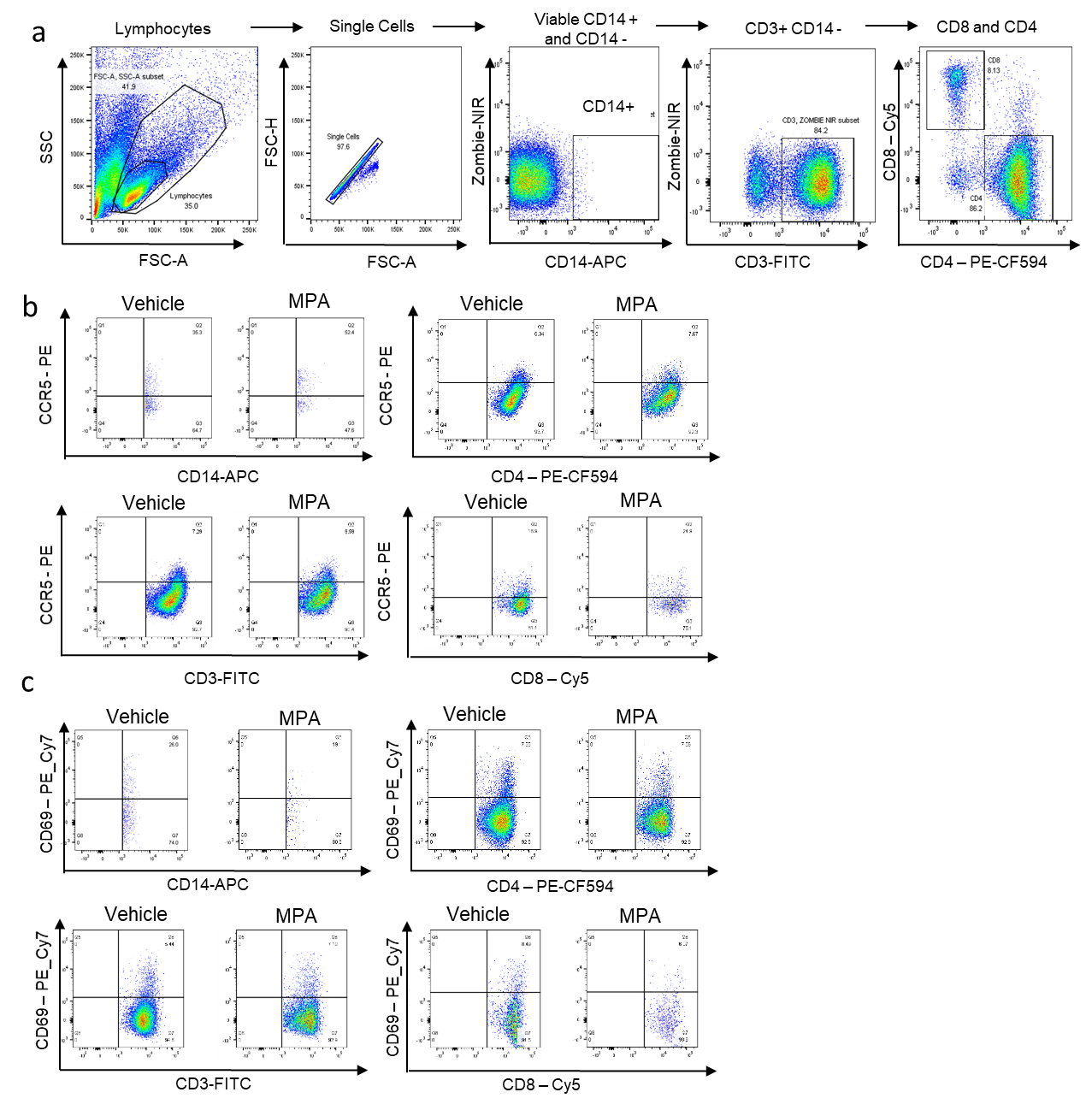
**Supporting information**

**Fig A. Gating strategy employed in this study.** Total PBMCs were stained at 24 hours or 7 days for different cell surface receptors and were subsequently analysed by flow cytometry. a) Lymphocytes were gated from the total cellular population, after which only the single cell population was used for analysis. Thereafter viable CD14– and CD14+ cells were gated. For the CD14– population, cells were further gated for CD3+ cells. Thereafter CD3+CD14- populations were gated for CD8+ and CD4+ cells. Upon establishing the different cellular populations CCR5+ (b) and CD69+ (c) expression within these populations were determined. Gating for each population was determined using the appropriate MFO controls.

**Table A. Frequencies of PBMC leukocytes and cells expressing CD25 or CCR5 in PBMCs stimulated with 100 nM MPA versus control for 24 hours.**

| **Immune cell phenotype** | **Vehicle** | | **100 nM MPA** | | **100 nM NET** | |
| --- | --- | --- | --- | --- | --- | --- |
|  | **Average** | **SEM** | **Average** | **SEM** | **Average** | **SEM** |
| **CD3+ (%total)** | 72.91 | 3.34 | 72.11 | 3.61 | 72.90 | 3.40 |
| **CD4+ (%total)** | 58.81 | 3.05 | 58.06 | 2.71 | 58.50 | 2.98 |
| **CD8+ (%total)** | 33.39 | 2.72 | 33.96 | 2.46 | 33.83 | 2.71 |
| **CD4/CD8 ratio** | 1.87 | 0.25 | 1.78 | 0.21 | 1.83 | 0.24 |
| **CD3+CD25+** | 8.38 | 1.58 | **9.57** | 1.59 | 8.27 | 1.74 |
| **CD4+CD25+** | 16.18 | 1.84 | **18.41** | 2.00 | 15.73 | 2.38 |
| **CD8+CD25+** | 0.68 | 0.18 | **1.04** | 0.29 | 0.66 | 0.18 |
| **CD3+CCR5+** | 2.32 | 0.78 | **2.70** | 0.87 | 2.10 | 0.79 |
| **CD4+CCR5+** | 9.83 | 3.34 | 10.26 | 3.47 | 8.04 | 2.26 |
| **CD8+CCR5+** | 2.94 | 1.07 | **3.52** | 1.20 | 2.75 | 1.23 |

Average frequency ± SEM indicated for n=10 independent PBMC donors. Statistical comparisons were carried out using a non-parametric Kruskal-Wallis one-way ANOVA with a Dunn’s post-test, but no significant changes were found. Where the fold changes (relative to Vehicle) were significantly different, as shown in Figure 2, the values in the table are in bold.

**Table B. Density of CD25 or CCR5 in PBMC leukocytes stimulated with 100 nM MPA versus control for 24 hours**

| **Immune cell phenotype** | **Vehicle** | | **100 nM MPA** | | **100 nM NET** | |
| --- | --- | --- | --- | --- | --- | --- |
|  | **Average** | **SEM** | **Average** | **SEM** | **Average** | **SEM** |
| **CD3+CD25+** | 4606 | 638.3 | **4916** | 641.9 | 4574 | 595.6 |
| **CD4+CD25+** | 2761 | 216.0 | **2944** | 184.3 | 2836 | 256.3 |
| **CD8+CD25+** | 2973 | 642.6 | 2531 | 338.0 | 2708 | 565.9 |
| **CD3+CCR5+** | 26214 | 817.5 | 27214 | 879.8 | 26531 | 957.0 |
| **CD4+CCR5+** | 9714 | 599.4 | 9660 | 585.1 | 9526 | 645.7 |
| **CD8+CCR5+** | 21227 | 3156.0 | **22071** | 3280.0 | 20459 | 3513.0 |

Average MFI ± SEM indicated for n=10 independent PBMC donors. Statistical comparisons were carried out using a non-parametric Kruskal-Wallis one-way ANOVA with a Dunn’s post-test, but no significant changes were found. Where the fold changes (relative to Vehicle) were significantly different, as shown in Figure 2, the values in the table are in bold.

**Table C. Frequencies of PBMC leukocytes and cells expressing CD69 or CCR5 in PBMCs stimulated with 100 nM MPA versus control for 7 days**

| **Immune cell phenotype** | **Vehicle** | | **100 nM MPA** | |
| --- | --- | --- | --- | --- |
|  | **Average** | **SEM** | **Average** | **SEM** |
| **CD3+ (%total)** | 79.70 | 3.57 | 73,13 | 3.904 |
| **CD4+ (%total)** | 63.43 | 4.629 | 67.43 | 4.134 |
| **CD8+ (%total)** | 27.35 | 4.025 | **21.37** | 4.374 |
| **CD14+ (%total)** | 36.81 | 13.52 | 35.05 | 13.19 |
| **CD4/CD8 ratio** | 3.16 | 0.94 | **4.84** | 1.28 |
| **CD3+CD69+** | 5.63 | 0.9596 | 6.16 | 0.5296 |
| **CD4+CD69+** | 6.84 | 0.917 | 9.44^a^ | 1.316 |
| **CD8+CD69+** | 6.38 | 0.9681 | 6.33 | 0.822 |
| **CD14+CD69+** | 29.98 | 1.725 | **19.19^***^** | 1.807 |
| **CD3+CCR5+** | 23.44 | 5.23 | 32.24 | 4.058 |
| **CD4+CCR5+** | 21.03 | 4.466 | 28.98 | 3.554 |
| **CD8+CCR5+** | 47.55 | 8.435 | 53.15 | 6.574 |
| **CD14+CCR5+** | 72.06 | 8.928 | 79.60 | 5.143 |

Average frequency ± SEM indicated for n=8 independent PBMC donors. Statistical comparisons were carried out using parametric unpaired t tests (*) or non-parametric Mann-Whitney (^a^). Significant differences are indicated by */^a^, ** and *** denoting p<0.05, p<0.01 and p<0.001, respectively. Where the fold changes (relative to Vehicle) were significantly different, as shown in Figure 3, the values in the table are in bold.

**Table D. Density of CD69 or CCR5 in PBMC leukocytes stimulated with 100 nM MPA versus control for 7 days**

| **Immune cell phenotype** | **Vehicle** | | **100 nM MPA** | |
| --- | --- | --- | --- | --- |
|  | **Average** | **SEM** | **Average** | **SEM** |
| **CD3+CD69+** | 4178,00 | 431,60 | 4023,50 | 340,00 |
| **CD4+CD69+** | 2170,75 | 234,00 | **2328,38** | 240,20 |
| **CD8+CD69+** | 4972,75 | 550,60 | 4499,50 | 318,60 |
| **CD14+CD69+** | 4202,75 | 122,00 | 3725,00 | 403,20 |
| **CD3+CCR5+** | 2609,25 | 142,70 | **3551,38^**^** | 245,00 |
| **CD4+CCR5+** | 2496,38 | 104,00 | **3533,63^**^** | 278,50 |
| **CD8+CCR5+** | 1193,38 | 146,90 | **1650,50** | 263,50 |
| **CD14+CCR5+** | 5203,88 | 1223,00 | 4803,25 | 1012,00 |

Average MFI ± SEM indicated for n=8 independent PBMC donors. Statistical comparisons were carried out using parametric unpaired t tests. Significant differences are indicated by *, ** and *** denoting p<0.05, p<0.01 and p<0.001, respectively. Where the fold changes (relative to Vehicle) were significantly different, as shown in Figure 3, the values in the table are in bold.
